# Burn Resuscitation

**DOI:** 10.1186/1757-7241-19-69

**Published:** 2011-11-11

**Authors:** Frederick W Endorf, David J Dries

**Affiliations:** 1 The Burn Center, Regions Hospital, 640 Jackson Street, St. Paul, MN 55101, USA; 2 Department of Surgery, Regions Hospital, 640 Jackson Street, St. Paul, MN 55101, USA

**Keywords:** Burn resuscitation, Crystalloid, Colloid

## Abstract

Fluid resuscitation following burn injury must support organ perfusion with the least amount of fluid necessary and the least physiological cost. Under resuscitation may lead to organ failure and death. With adoption of weight and injury size-based formulas for resuscitation, multiple organ dysfunction and inadequate resuscitation have become uncommon. Instead, administration of fluid volumes well in excess of historic guidelines has been reported. A number of strategies including greater use of colloids and vasoactive drugs are now under investigation to optimize preservation of end organ function while avoiding complications which can include respiratory failure and compartment syndromes. Adjuncts to resuscitation, such as antioxidants, are also being investigated along with parameters beyond urine output and vital signs to identify endpoints of therapy. Here we briefly review the state-of-the-art and provide a sample of protocols now under investigation in North American burn centers.

## Introduction

One of the most challenging aspects of caring for burned patients is the acute resuscitation. The profound inflammatory response generated by a burn far surpasses that seen in trauma or sepsis, and the resultant fluid needs can be extreme. There is a large and ever-increasing body of research devoted to refining strategies for acute burn resuscitation, and this article attempts to summarize some the most important recent findings in the field.

After treating victims of the infamous Coconut Grove fire in 1942, Cope and Moore first postulated that burn resuscitation needs may have contributions from both the patient's body weight and the size of their burn [1]. Baxter and Shires later built on this knowledge, using canine and human data, to specifically measure fluid requirements by weight and total body surface area (%TBSA). Their formula of 3.5 to 4.5 ml of lactated Ringers per %TBSA per kilogram became known as the Parkland formula after the Dallas medical complex in which their experiments took place [2]. Although the Parkland formula is still the most commonly employed resuscitation formula worldwide, it is far from a perfect solution.

Ongoing research focuses on refining existing formulas to prevent complications of over-resuscitation. This includes devising novel means for titrating resuscitation, such as nurse-driven or computer-driven protocols. The composition of the fluids used in resuscitation has generated significant interest, with a particular focus on colloids and hypertonic saline. Pharmaceutical therapies that attempt to down regulate the inflammatory response such as vitamin C may have a role in acute resuscitation. Likewise, investigators are proposing the use of adjuncts such as plasmapheresis to remove inflammatory mediators from the bloodstream during resuscitation. The final topic inviting significant scrutiny is outcomes of resuscitation, both choosing the most appropriate outcomes to use and how to best measure these outcomes in clinical practice.

### Resuscitation Volumes

The Parkland formula is simple to calculate and has a long record of widespread use in the burn community. This has not stopped researchers from frequently examining the efficacy of this formula, and also determining whether practitioners of this resuscitation formula are using it correctly.

The most consistent criticism of the Parkland formula is that patients tend to receive more fluid than the formula would have predicted based on the patient's weight and %TBSA. Whether this is an inherent flaw of the formula or user error by practitioners is debated, but it is clear that fluid volumes used in resuscitation are higher than historical controls. This phenomenon was described by Pruitt as "fluid creep [3]." Engrav et al. in a multi-center study in 2000 reviewed resuscitations in 50 patients and confirmed that 58% of patients required more fluid than predicted by the Parkland formula, compared to 12% exceeding predicted values in Baxter's original work [4]. Friedrich et al. continued this investigation in 2004, comparing recent patients at their center to those from the 1970's, and found that fluid requirements during resuscitation had doubled during that period [5]. In a related study, they correlated this change with a marked increase in narcotic pain medication usage during the resuscitation period. They theorized that the vasodilatory effects of opioid medications may cause relative hypotension, in turn necessitating increased fluid administration [6]. This finding was later replicated by Wibbenmeyer et al. who showed a strong correlation between opioid equivalents received in the first 24 hours post-burn and the fluid volumes during that same period [7]. Even at Parkland medical center, Blumetti et al. found that 48% of patients were receiving higher than predicted fluid volumes [8]. Despite growing awareness of this "fluid creep," Cartotto et al. found that recent patients at their center were continuing to receive an average of 6.3 ml/kg/%TBSA during resuscitation, with 76% getting more than the 4.3 ml/kg/%TBSA in the original Baxter work [9].

### Resuscitation Protocols

One factor playing into the trend of higher resuscitation volumes may be inadequate titration of fluids by physicians. Cancio et al. reviewed their experience with resuscitations using the modified Brooke formula, which predicts 2 ml/kg/%TBSA fluid volumes. An important finding was that clinicians directing the resuscitation were significantly less likely to reduce the rate of fluid infusion when urine output was high than they were to increase fluid rates when urine output was low [10]. In an effort to reduce the dependence on clinical decision-making, several centers have experimented with standardized algorithms derived from hourly urine output. Nurse-driven resuscitation protocols may ameliorate differences in resuscitation due to practitioner experience. Established algorithms may have better reinforcement of downward titration of fluid volumes when urine output is high, and may even allow for reductions in infusion rates when urine output is adequate. Jenabzadeh et al. used a nurse-driven protocol and showed a significant decrease in fluid volumes during resuscitation and a dramatic decrease in the incidence of abdominal compartment syndrome [11].

To further remove the element of human error, some centers have been incorporating computer-based resuscitation algorithms. Salinas et al. described a computer model for burn resuscitation that was utilized in the resuscitation of 32 burn patients that were compared to historical controls. Their protocol resulted in less 24-hour and 48-hour crystalloid volumes, and less total crystalloid volumes while in the intensive care unit. Volumes by body weight and by burn area were also significantly less for the computer resuscitation group. The computer protocol also helped patients to more effectively meet hourly goals for urine output [12]. In a follow-up study, Salinas et al. analyzed how practitioners used the computer program's recommendations and found that they followed the computer recommendations 83.2% of the time. Reasons given for refusing the computer recommendations were when clinicians felt the recommended fluid increase was either excessive or inadequate, or if a patient was hypotensive [13].

### Colloid

Historically, prevailing opinion had been that using colloid in the first 24 hours of resuscitation was contraindicated. It was thought that colloid would pass through the "leaky" capillaries in burn shock and exert an osmotic pull, drawing even more fluid into the interstitial space and worsening burn edema. However, investigators more recently are advocating the use of colloid in burn resuscitation, even in the first 24 hours.

Lawrence et al. performed a retrospective review of 52 burn patients with greater than 20% TBSA burns. Twenty-six of these patients received albumin during their resuscitation, and 26 only crystalloid. As part of an institutional resuscitation algorithm, patients requiring more fluid volumes than predicted by the Parkland formula changed to an arm of the algorithm in which they got a third of their hourly fluid volumes as 5% albumin, with the other two-thirds given as lactated Ringer's (LR) solution. After colloid infusion began, patients quickly returned to predicted fluid rates and stayed at those lower volumes for the remainder of their resuscitation. Neither the colloid nor crystalloid group had any patients with abdominal compartment syndrome, though the colloid patients had more extremity escharotomies, likely related to their larger average burn size [14]. In a previous study from the same center, Cochran et al. performed a case-control analysis of large burns (>20% TBSA) that either did or did not receive albumin during their resuscitation. Not only was albumin not harmful, it actually conferred a mortality benefit in their study that confirmed on multivariate analysis [15]. This phenomenon seems to hold in pediatric patients as well. The same group examined 53 pediatric patients with greater than 15% TBSA burns and found that patients with higher than predicted fluid volumes "normalized" with albumin administration. Again, there were no cases of abdominal compartment syndrome, and in the pediatric population there was no difference in the incidence of extremity or torso escharotomy. The albumin group did have a longer length of stay, again likely related to larger burn size and a higher rate of inhalation injury [16]. The use of artificial colloids in burn resuscitation has also generated significant interest. Vlachou et al. randomized 26 adult patients to either a purely crystalloid resuscitation or one substituting 6% hydroxyethylstarch (HES) for one third of the predicted crystalloid volume. They found that patients in the HES arm required less overall fluid volumes in the first 24 hours and subsequently had less increase in body weight. They also measured C-reactive protein as a putative marker for inflammation and found lower values in the HES group [17]. However, caution should be used with higher concentrations of HES. Bechir et al. studied a 10% HES solution versus crystalloid in 30 burned patients and found a trend toward higher rates of renal failure and higher mortality, though neither reached statistical significance [18].

### Hypertonic Fluids

In an attempt to prevent overresuscitation, some investigators have also started using hypertonic saline, alone or in combination with colloid. Belba et al. performed a prospective, randomized study of 110 burned patients, 55 of whom were resuscitated with LR according to the Parkland formula for adults and the Shriner's formula for children. The other 55 patients received a hypertonic saline lactate solution containing sodium (250 mEq/L) and lactate (120 mEq/L). The hypertonic group needed higher fluid rates initially, but both groups decreased to less than predicted by the Parkland formula over the first 24 hours. The hypertonic group used less fluid overall than the isotonic fluid group, but this difference was not statistically significant [19]. Using a hypertonic solution during resuscitation may also lower the risk of abdominal compartment syndrome. Oda et al. reviewed 36 patients with greater than 40% TBSA burns, 14 of whom were resuscitated using a hypertonic lactated saline solution and 22 with LR solution. The hypertonic solution was given in a staggered fashion, starting with a solution of sodium (300 mEq/L), chloride (88 mEq/L), and lactate (212 mEq/L). This was tapered down gradually, ending 48 hours after the burn with a solution of sodium (150 mEq/L), chloride (102 mEq/L), and lactate (48 mEq/L). They found that two of 14 patients in the hypertonic group developed abdominal compartment syndrome versus 11 of 22 in the LR group [20].

### Antioxidants

The extensive inflammation seen in burn injury causes free oxygen radical release, which worsens vascular permeability and subsequently causes significant peripheral edema. The loss of fluid into the interstitium results in higher fluid needs during resuscitation. It is though that the use of antioxidants during resuscitation may help scavenge these free radicals and attenuate vascular permeability.

Tanaka et al. compared two groups of patients, 18 that were resuscitated with LR alone, and the other 19 patients receiving LR plus high dose ascorbic acid (vitamin C, 66 mg/kg/hr). They found average fluid needs of 3 ml/kg/%TBSA in the vitamin C group versus 5.5 ml/kg/%TBSA in the group resuscitated with LR alone. In addition, the vitamin C group had fewer ventilator days [21]. Kahn et al. performed a retrospective review of 33 patients, 17 of whom had high-dose (66 mg/kg/hr) vitamin C plus LR, and 16 who had LR alone. They also found lower average fluid volumes in the vitamin C plus LR group (5.3 ml/kg/%TBSA) compared to the LR group (7.1 ml/kg/%TBSA). There was no difference in outcomes in their study, but also no difference in complications. They conclude that vitamin C is a safe adjunct for decreasing fluid volumes in the first 24 hours of burn resuscitation [22].

In a murine model, Constantini et al. experimented with the use of pentoxifylline (PTX) after burns. After a 30% steam burn, PTX in saline alone was injected intraperitoneally into one group of mice and saline alone into another group. The group with PTX had decreased intestinal permeability and inflammation. In a secondary finding, they also noted a decreased incidence of acute lung injury in the PTX group. Although there are no existing human trials with PTX in burn resuscitation, it may have promise as an antioxidant immune modulator during acute resuscitation [23].

### Plasmapheresis

In addition to using antioxidants, some centers have looked at using mechanical removal of inflammatory mediators from the bloodstream. Klein et al. reviewed the use of plasma exchange at their institution over a 5-year period, in which 37 burn patients underwent plasma exchange during their acute resuscitation, seven of whom received two treatments for a total of 44 plasma exchanges. These were severe burns with a mean %TBSA of 48.6%, and 73% of the patients had associated inhalation injury. There was no protocol for initiation of plasma exchange, but it was often prompted by reaching twice the predicted Parkland formula resuscitation volume. Average time to initiation of plasma exchange was 17 hours, and the average duration of therapy was 2.4 hours. Albumin (5%) was primarily used as replacement fluid, unless the patient had low fibrinogen or abnormal clotting factors, in which case fresh frozen plasma (FFP) was used. They found that plasma exchange decreased crystalloid administration by 28.3%. When adjusted for patient weight and %TBSA, the average post-exchange fluid resuscitation volumes dropped by 40%. After plasma exchange, the hourly fluid administration rates never returned to preexchange levels in any patient [24].

Neff et al. performed a retrospective case-control study of 40 patients over a two-year period, all of whom had greater than 20% TBSA burns. Twenty-one of these patients underwent plasma exchange as part of their resuscitation, and they were matched with 19 contemporaneous controls. Plasma exchange was triggered by fluid volumes of 1.2 times that predicted by the Parkland formula, or by continued low urine output or hypotension in the face of escalating fluid rates. They found several physiologic benefits with plasma exchange, including a 24% increase in mean arterial pressure (MAP), a 400% increase in urine output, and a 25% reduction in intravenous fluid resuscitation rates need to maintain vital signs and urine output goals. Lactate levels also decreased, and they noted that an elevated admission lactate independently predicted the need for eventual plasma exchange [25].

### Outcomes of resuscitation and monitoring

The second challenge in management of burn resuscitation is determination of optimal clinical endpoints. Traditionally, urine output has been used as the primary gauge of tissue perfusion during acute resuscitation. Greenhalgh recently published findings from a survey of American Burn Association (ABA) and International Society for Burn Injuries (ISBI) members regarding various topics in resuscitation. Respondents (94.9%) used urine output as a major index of successful resuscitation, with 22.7% using other monitors [26]. Despite its widespread use, urine output is not generally viewed as a perfect measure of overall tissue perfusion. Concern has also been raised about effectiveness of other conventional parameters including blood pressure, heart rate, and central venous pressure. There are several techniques being examined that aim to more accurately measure peripheral perfusion, and allow better titration of fluid volumes on a real-time basis.

Parameters derived from transcardiopulmonary thermodilution using the PiCCO system have shown good correlation with values from a conventional pulmonary artery catheter in burned patients [27]. The use of this system has also confirmed the hyperdynamic physiologic response in pediatric patients with large burns [28]. However, there exist no studies showing any influence of this system on outcomes during acute burn resuscitation.

Jeng et al. studied four patients with severe (average 58% TBSA) burns and shock, using a multisensory probe with three transducers. One transducer was placed in the subcutaneous tissue in a representative second-degree burn, another transducer in the stomach via a tonometric gastric tube, and a third into the bloodstream through a single-lumen femoral line. They then used these transducers to measure tissue pH, CO_2_, and PaO_2_. They also simultaneously charted urine output, mean arterial pressure (MAP), and serum lactate. Third, these investigators measured burn wound perfusion with laser Doppler imaging and correlated the previous variables with observed changes in burn wound perfusion. Although changes in all variables were associated with changes in Doppler perfusion, they found that tissue pH and CO_2_, as well as gastric CO_2_, had the closest temporal relationship to changes in peripheral perfusion. Urine output, MAP, and lactate did change over time but tended to lag behind changes in peripheral tissue perfusion measured with laser Doppler imaging [29].

Though lactate may not give "real-time" information about resuscitation success, it does predict morbidity and mortality in burned patients. Cochran et al. reviewed 128 patients with an average of 41.7% TBSA and measured base deficit and lactate levels at 6-hour intervals. They found that the non-survivors had higher lactates at admission, 12, 18, and 24 hours than the group of survivors. Elevation of lactate in the first 48 hours was an independent predictor of mortality, but they were unable to demonstrate a specific threshold for clinical use. The authors caution that treatment should not be withheld based on any individual laboratory value [30].

## Conclusion

Burn resuscitation continues to be a complex and challenging phase of care for burn patients. The long-running trend of increases in crystalloid fluid volumes is now recognized by practitioners, and efforts are being made to reduce excess fluid administration when possible. Refinement in resuscitation protocols, as well as multiple adjunctive therapies, may help reduce excess crystalloid administration. Finding a more accurate measure of resuscitation success could allow better and faster responses to physiologic changes.

The best consensus statement available comes from the *American Burn Association *in 2008. While there has been significant work examining alternatives to standard resuscitation practices, the ultimate consensus report retains emphasis on a crystalloid-based resuscitation utilizing 2-4 mL/kg/body weight/%TBSA during the first 24 hours. Fluid should be isotonic and titrated to maintain urine output of 0.5 to 1.0 mL/kg/hr in adults and 1.0-1.5 mL/kg/hr in children. Children may require additional fluid due to maintenance requirements. Incremental volume administration may be necessary in patients with significant full-thickness injury, delayed resuscitation or smoke inhalation (Additional File 1, Box 1) [31].

While these basic consensus guidelines may not reflect the variety of research carried on in burn resuscitation in recent years, this does reflect a safe starting point, particularly for practitioners in centers without extensive experience in burn resuscitation attempting to stabilize a thermal injury victim prior to transfer to a burn center.

With other burn centers in the United States, we are studying approaches to limit crystalloid administration with protocol-based use of vasoactive drugs and colloids in patients failing to respond to the initial resuscitation prescription. Crystalloid administration is capped at 100 mL of fluid/kg with second and third degree injury in our practice. When this physical limit is reached, a transition is made to colloids regardless of the time since injury. While this approach is designed and originally intended for adult patients, we now use it in all age groups (Additional File 2, Figure S1 and Additional File 3, Figure S2).

Traditional practice in the resuscitation of surgical patients included an automatic increase in fluid administration for hypotension with strict avoidance of vasoactive drugs, particularly norepinephrine, and diuretic administration. While we continue to emphasize careful evaluation of urine output and vital signs, vasopressin, norepinephrine or dobutamine may be employed after initial response to fluid administration is evaluated. The hypotensive patient with acceptable central venous pressure may receive vasopressin or norepinephrine. The patient with elevated blood pressure and central venous pressure may receive furosemide and dobutamine in addition to decreased resuscitation fluids. This approach incorporating more resuscitation options should be done in consultation with a center having expertise in burn management.

Please refer to Additional File 4, Box 2 for a summary of key points.

## Competing interests

The authors declare that they have no competing interests.

## Authors' contributions

FE performed the literature review and wrote the initial draft of the manuscript. DD conceived of the study and edited and rewrote portions of the manuscript. All authors read and approved the final manuscript.

## Author Information

Frederick W. Endorf, MD is Staff Surgeon at Regions Hospital, the Level I Trauma and Burn Center, in St. Paul, Minnesota, USA. He is also Clinical Assistant Professor of Surgery at the University of Minnesota.

David J. Dries, MSE, MD, FACS, FCCM, FCCP is the Assistant Medical Director of Surgical Care for HealthPartners Medical Group and Division Head for Surgery at Regions Hospital, the Level I Trauma and Burn Center, in St. Paul, Minnesota, USA. He is also Professor of Surgery, Professor of Anesthesiology and Clinical Adjunct Professor of Emergency Medicine at the University of Minnesota. Dr. Dries also holds the *John F. Perry, Jr. Chair of Trauma Surgery *at the University of Minnesota.

## Supplementary Material

Additional file 1 **Box 1. American Burn Association Consensus Guidelines- 2008**. Basic consensus guidelines in burn resuscitation. Click here for file

Additional file 2 **Figure S1. Nurse Driven Resuscitation Protocol**. This detailed resuscitation approach incorporates vasoactive drugs, fresh frozen plasma, and albumin, and is designed for adults with greater than 20% Body Surface Area Burn (BASB) or young adults as defined. The starting point for fluid resuscitation is the standard Parkland formula utilizing lactated Ringer's at 2 to 4 mL/kg/% burn. Subsequent therapy is titrated based on urine output and vital signs. *In large burns, our crystalloid limit of 100 mL/kg means that transition to colloids or vasoactive drugs occurs well before the conclusion of 24 hours of resuscitation*. Note that this protocol is designed for utilization by the bedside nurse. However, if vasoactive drugs or colloids are considered, burn unit faculty are immediately engaged. Click here for file

Additional file 3 **Figure S2. Colloid Protocol and Pressor Protocol**. Where patients are not successfully resuscitated using a simple crystalloid protocol, options include administration of fresh frozen plasma or albumin, which can be very valuable in children as dilution of serum albumin with crystalloids can rapidly occur. Vasoactive drugs are utilized in conjunction with a central venous catheter and measurement of central venous pressure. Bladder pressures are also monitored via the urinary catheter to identify intraabdominal hypertension and minimize the risk of intraabdominal compartment syndrome in patients receiving large resuscitation volume [32-34]. Finally, our unit continues to use fresh frozen plasma (FFP) as a part of our resuscitation strategy. This is given when crystalloid volumes exceed 100 mL/kg (see section of Additional File 3, Figure S2 marked "Colloid Protocol"). FFP is administered at 0.5 mL/kg/%TBSA, transfused over 8 hours. We recognize that this is controversial and also use albumin in selected patients. Click here for file

Additional file 4 **Box 2. Key Points**. Resuscitation options in the burned patient. Click here for file
