# Supplementary material for: Burn Resuscitation
Source: Scand J Trauma Resusc Emerg Med. 2011 Nov 11;19:69. doi: 10.1186/1757-7241-19-69 (PMC3226577; doi:10.1186/1757-7241-19-69)
Supplement: Additional file 1 — Box 1. American Burn Association Consensus Guidelines- 2008. Basic consensus guidelines in burn resuscitation. [file 1757-7241-19-69-S1.DOC]

**Additional File 1, Box 1**

**American Burn Association Consensus Guidelines- 2008**

| - Adults and children with burns greater than 20% TBSA should undergo formal fluid resuscitation. |
| --- |
| - Crystalloid 2-4 mL/kg body weight/%TBSA during the first 24 hours (Lactated Ringer’s commonly used. |
| - Fluid titrated to urine output of 0.5-1.0 mL/kg/hr (adults) and 1.0-1.5 mL/kg/hr (children). |
| - Children require maintenance fluids along with burn resuscitation. |
| - Increased volume requirements:   - full-thickness injuries   - inhalation injury   - delay in resuscitation |
