# Supplementary material for: Burn Resuscitation
Source: Scand J Trauma Resusc Emerg Med. 2011 Nov 11;19:69. doi: 10.1186/1757-7241-19-69 (PMC3226577; doi:10.1186/1757-7241-19-69)
Supplement: Additional file 2 — Figure S1. Nurse Driven Resuscitation Protocol. This detailed resuscitation approach incorporates vasoactive drugs, fresh frozen plasma, and albumin, and is designed for adults with greater than 20% Body Surface Area Burn (BASB) or young adults as defined. The starting point for fluid resuscitation is the standard Parkland formula utilizing lactated Ringer's at 2 to 4 mL/kg/% burn. Subsequent therapy is titrated based on urine output and vital signs. In large burns, our crystalloid limit of 100 mL/kg means that transition to colloids or vasoactive drugs occurs well before the conclusion of 24 hours of resuscitation. Note that this protocol is designed for utilization by the bedside nurse. However, if vasoactive drugs or colloids are considered, burn unit faculty are immediately engaged. [file 1757-7241-19-69-S2.DOC]

**Additional File 2, Figure 1-Nurse Driven Resuscitation Protocol**

Young Adult (≥ 40 kg and > 12 yo) or Adult Acute Burn Patient with > 20% BSAB

**WEIGHT: ­­­­___ kg**

**Vitals unstable:**

**HR > 140 or**

**MAP < 60 or**

**SBP <90**

**STEP ONE**

Begin fluid resuscitation rate as ordered by attending MD.

Then measure urine output (UOP) hourly.

**CALL ATTENDING**

& consider

Pressor Protocol

***Figure 2***

Vital Signs Stable: HR<140, SBP>90, MAP>60

UOP < 0.3 mL/kg/hr

UOP 0.3-0.5 mL/kg/hr

UOP > 0.5 mL/kg/hr

***See Figure 2 for Weight and UOP Table***

See Pressor Protocol

***Figure 2***

Leave IVF at current rate

↓ IVF by 10%

REPEAT **STEP ONE** EVERY HOUR UNTIL:

If total IVF > 100mL/kg

within first 24 hrs,

start FFP resuscitation

***Figure 2***

Pt is 24 hrs post burn

UOP < 0.3 mL/kg/hr for 2 hrs despite

an ↑ IVF

Start FFP Resuscitation

(if not given already)

**FFP Resuscitation:**

FFP at 0.5 mL/kg/%BSAB over 8 hrs

Check foley, assess breath sounds, vital signs and bladder pressure.

IVF Maintenance Rate is reached & held for 2 hrs

**No**

Switch to

**D5 ½NS + 20 mEq KCl/L**

at Maintenance Rate

and

LR at a rate so that:

MIVF + LR = current IVF rate

**CALL ATTENDING**

& consider

Colloid Protocol vs. Pressor Protocol

(see below)

***Figure 2***

**Yes**

Fluid resuscitation is **COMPLETE**

Switch to

**D5 ½NS + 20 mEq KCl/L**

at Maintenance Rate

Repeat **STEP ONE**

& titrate LR to off.

If pt again develops oliguria or hemodynamic instability,

**CALL ATTENDING**
