# Supplementary material for: Burn Resuscitation
Source: Scand J Trauma Resusc Emerg Med. 2011 Nov 11;19:69. doi: 10.1186/1757-7241-19-69 (PMC3226577; doi:10.1186/1757-7241-19-69)
Supplement: Additional file 3 — Figure S2. Colloid Protocol and Pressor Protocol. Where patients are not successfully resuscitated using a simple crystalloid protocol, options include administration of fresh frozen plasma or albumin, which can be very valuable in children as dilution of serum albumin with crystalloids can rapidly occur. Vasoactive drugs are utilized in conjunction with a central venous catheter and measurement of central venous pressure. Bladder pressures are also monitored via the urinary catheter to identify intraabdominal hypertension and minimize the risk of intraabdominal compartment syndrome in patients receiving large resuscitation volume [32-34]. Finally, our unit continues to use fresh frozen plasma (FFP) as a part of our resuscitation strategy. This is given when crystalloid volumes exceed 100 mL/kg (see section of Additional File 3, Figure S2 marked "Colloid Protocol"). FFP is administered at 0.5 mL/kg/%TBSA, transfused over 8 hours. We recognize that this is controversial and also use albumin in selected patients. [file 1757-7241-19-69-S3.DOC]

**Additional File 3, Figure 2-Colloid Protocol and Pressor Protocol**

**Colloid Protocol**

**(FFP/Albumin)**

**Pressor Protocol**

If unable to wean IVF rate

**OR**

UOP < 0.3 mL/kg/hr for 2 hrs

SBP > 90 **&** MAP > 60

**&**

UOP < 0.3 mL/kg/hr for 2 hrs

SBP < 90 **or** MAP < 60

**Vasopressin**

UOP < 0.3 mL/kg/hr for 2 hrs

CVP < 8

CVP 8-16

**Call attending** to discuss starting

**FFP Resuscitation**

(if hasn’t been given already)

**or**

50 mL of 25% Albumin Q6H for 48 hrs

(Both in addition to current IVF rate)

CVP < 8

CVP > 16

CVP > 16

CVP 8-16

↑ IVF by 10% and

Repeat **STEP ONE**

***Figure 1***

↑ IVF by 10%

+ 5% Alb bolus.

Then repeat

**STEP ONE**

***Figure 1***

Repeat **STEP ONE**

***Figure 1***

**Vasopressin**

**Lasix +/-**

**Dobutamine**

**Levophed**

**Dobutamine**

Wean all medications as tolerated.

Repeat **STEP ONE**.

***Figure 1***

**All medications must prompt a call to**

**the Burn Attending:**

- **Dobutamine:** 2-20 mcg/kg/min IV
- **Lasix:** 20 mg IV
- **Levophed:** 2-12 mcg/kg/min IV
- **Vasopressin:** 2.4 units/h IV
- **Bolus =** 5% Alb 250 mL

| **Weight (kg)** | **UOP Range (mL/h)** |
| --- | --- |
|  |  |
| ≥100 | 30-50 |
| 85-99 | 25-45 |
| 75-84 | 25-40 |
| 65-74 | 20-35 |
| 55-64 | 20-30 |
| 45-54 | 15-25 |
| 40-44 | 15-20 |
