# Supplementary material for: Burn Resuscitation
Source: Scand J Trauma Resusc Emerg Med. 2011 Nov 11;19:69. doi: 10.1186/1757-7241-19-69 (PMC3226577; doi:10.1186/1757-7241-19-69)
Supplement: Additional file 4 — Box 2. Key Points. Resuscitation options in the burned patient. [file 1757-7241-19-69-S4.DOC]

**Additional File 4, Box 2**

**Key Points**

| 1. Attempts should be made to minimize over-resuscitation in the burn patient |
| --- |
| 2. Nurse-driven or computer-driven protocols help to more accurately titrate fluid |
| 3. Colloid should be used in acute resuscitation when crystalloid exceeds predicted volumes |
| 4. Antioxidants show promise in reducing resuscitation volumes |
| 5. Plasma exchange is useful in patients not responding to conventional fluid resuscitation |
| 6. Tissue perfusion may be a more accurate endpoint of resuscitation than urine output, though urine output is still most commonly used |
| 7. Vasoactive medications may safely be used during resuscitation in select patients after properly ensuring adequate fluid resuscitation |
